# Efficacy comparison of immune combination therapies in subgroups for advanced hepatocellular carcinoma patients: Systematic review and network meta-analysis

**DOI:** 10.1371/journal.pone.0306869

**Published:** 2024-07-22

**Authors:** Yani Wang, Wanyee Lau, Yafei Li, Yichen Tian, Yongrong Lei, Feng Xia, Jianhua Wang

**Affiliations:** 1 Key Laboratory of Biorheological Science and Technology, Ministry of Education, College of Bioengineering, Chongqing University, Chongqing, China; 2 Faculty of Medicine, Prince of Wales Hospital, Chinese University of Hong Kong, Shatin, New Territories, Hong Kong, SAR, China; 3 Department of Epidemiology, College of Preventive Medicine, Army Medical University (Third Military Medical University), Chongqing, China; 4 Key Laboratory of Hepatobiliary and Pancreatic Surgery, Institute of Hepatobiliary Surgery, Southwest Hospital, the First Hospital Affiliated to AMU (Southwest Hospital), Chongqing, China; Seoul National University College of Pharmacy, REPUBLIC OF KOREA

## Abstract

**Background:**

There is a lack of precision in the immunotherapy strategy tailored for patients exhibiting diverse clinical characteristics. This study aims to employ a rigorous network meta-analysis (NMA) approach to systematically evaluate the effectiveness of immune-combination therapies among patients with advanced hepatocellular carcinoma, taking into account their varying clinico-characteristics.

**Methods:**

Studies were retrieved from PubMed, Embase, Cochrane Library, and Web of Science databases. The included first-line phase III studies were categorized into three types: immunotherapy combined with anti-angiogenetic agents, immunotherapy combined with tyrosine kinase inhibitors, and dual immunotherapy, with sorafenib serving as the control group. The primary endpoint used to assess efficacy was overall survival (OS), facilitating a comparative analysis among the three treatment modalities. Furthermore, subgroup analyses were conducted to evaluate the varying effectiveness for patients with diverse clinico-characteristics. Secondary outcome measures included progression-free survival, objective response rate, and toxicity assessment.

**Results:**

A total of 6 studies were included in the NMA, encompassing a cohort of 3840 patients. The results revealed that immunotherapy combined with anti-angiogenetic agents exhibited a significantly enhanced therapeutic effect in terms of improving OS compared to sorafenib (HR = 0.61, 95% CrI, 0.42–0.90). Furthermore, based on various clinicopathological features, this combination therapy demonstrated superior OS responses in specific patient subgroups: BCLC C (HR = 0.63, 95% CrI, 0.42–0.93), ECOG 1 (HR = 0.57, 95% CrI, 0.36–0.91), with extrahepatic spread (EHS) (HR = 0.59, 95% CrI, 0.37–0.92), alpha fetoprotein (AFP)<400ng/ml (HR = 0.56, 95% CrI, 0.33–0.94) and viral hepatitis positivity (HR = 0.56, 95% CrI, 0.39–0.77) (especially HBV (HR = 0.58, 95% CrI, 0.40–0.85)). Importantly, the advantage of this combination therapy was even more pronounced in patients with viral hepatitis positivity. Also, the adverse events associated with immunotherapy combined with antiangiogenic drugs were moderate.

**Conclusions:**

Immunotherapy combined with anti-angiogenetic agents could represent the most effective first-line intervention for achieving improved OS, particularly in patients with viral hepatitis positivity.

## Introduction

Hepatocellular carcinoma (HCC) represents the predominant type of primary liver cancer globally, accounting for approximately 80% of all reported cases [1]. The diagnosis of HCC often manifests in the intermediate to advanced stages of tumor progression, highlighting the paramount importance of implementing systemic therapeutic strategies. The initial approval of atezolizumab, administered concomitantly with bevacizumab, as a frontline treatment option for unresectable HCC in Europe has subsequently led to its widespread endorsement by international guidelines as the preferred choice for managing advanced HCC [2–4]. As a result, immune-combination targeted therapy has undergone rapid development, leading to significant advancements in the treatment of HCC. At the European Society for Medical Oncology 2022, phase III results were announced pertaining to the latest immune-combination targeted therapy approach for HCC. These results revealed positive outcomes in terms of the dual primary endpoints of overall survival (OS) and progression-free survival (PFS) in the CARES-310 study [5]. Notably, a substantial benefit was observed in the subgroup of patients infected with the hepatitis B virus (HBV), with hazard ratios (HRs) of 0.53 (95% confidence interval (CI): 0.41–0.68) for OS and 0.66 (95% CI: 0.50–0.87) for PFS, respectively. In the LEAP-002 study [6], while the improvements in OS and PFS did not meet the prespecified statistical criteria for significance, there was a demonstrated survival advantage in patients with extrahepatic metastases (EHS) (HR = 0.78, 95% CI: 0.63–0.95), α-fetoprotein (AFP) levels> 400ng/ml (HR = 0.67, 95% CI: 0.50–0.90), and hepatitis B virus (HBV) infection (HR = 0.75, 95% CI: 0.58–0.97).

The Food and Drug Administration (FDA) recently approved the combination of durvalumab and tremelimumab in 2022, establishing the first dual immunotherapy option as a front-line treatment for patients with advanced HCC. This approval signifies a significant milestone, as it offers a 22% reduction in the risk of mortality compared to sorafenib, with a hazard ratio of 0.78 and a p-value of 0.0035 [7]. Notably, the therapeutic response appears to be particularly favorable in patients with HBV positivity (HR = 0.64, 95%CI:0.48–0.86). This approval further presents a promising alternative for individuals who are not candidates for anti-vascular endothelial growth factor (VEGF) therapy.

Until now, there has been a notable scarcity of studies that have comprehensively evaluated the comparative efficacy of immune-combination therapies in treating patients with advanced HCC who possess diverse clinical characteristics. Although some general understanding of the subject matter exists, there is a lack of specific studies that have delved into the distinct differences among these therapeutic options when stratified across various subgroups. Therefore, it is imperative to conduct a thorough and systematic review, coupled with a network meta-analysis, of first-line treatment options for advanced HCC. This enables us to compare the effectiveness of immune combination therapies across different subgroups of pathological features, including immunotherapy combined with anti-angiogenetic agents, immunotherapy combined with tyrosine kinase inhibitors (TKIs), and dual immunotherapy. Additionally, it is essential to assess the efficacy of these treatment modalities in terms of OS, PFS and objective response rate (ORR). Moreover, the toxicity profile and incidence of treatment-related adverse events (TRAEs) must be meticulously evaluated and quantified.

## Materials and methods

The study has been reported in line with PRISMA [8] (Preferred Reporting Items for Systematic Reviews and Meta-Analyses) and AMSTAR [9] (Assessing the methodological quality of systematic reviews) Guidelines. The study was registered in PROSPERO with CRD42023425250.

### Searching strategy

The primary focus of this study was to conduct a meticulous and organized search pertaining to English literature and conference abstracts. We embarked on a comprehensive and systematic examination of pertinent articles that were published within the timeframe of January 2003 to December 2023. These articles were sourced from various reputable databases, including Embase, PubMed, Cochrane Library, and Web of Science. The principal keywords utilized in our search encompassed hepatocellular carcinoma; liver cancer Adult; HCC; and immunotherapy.

Furthermore, to ensure the inclusion of the most recent and pertinent findings, we also searched through the memoirs of the 2022 European Society for Medical Oncology and American Society of Clinical Oncology Meetings. This enabled us to capture the latest published results relevant to our study. A detailed outline of the comprehensive searching strategies is presented in S1 Table in S2 File.

### Criteria for selecting trials

**Fig 1:**
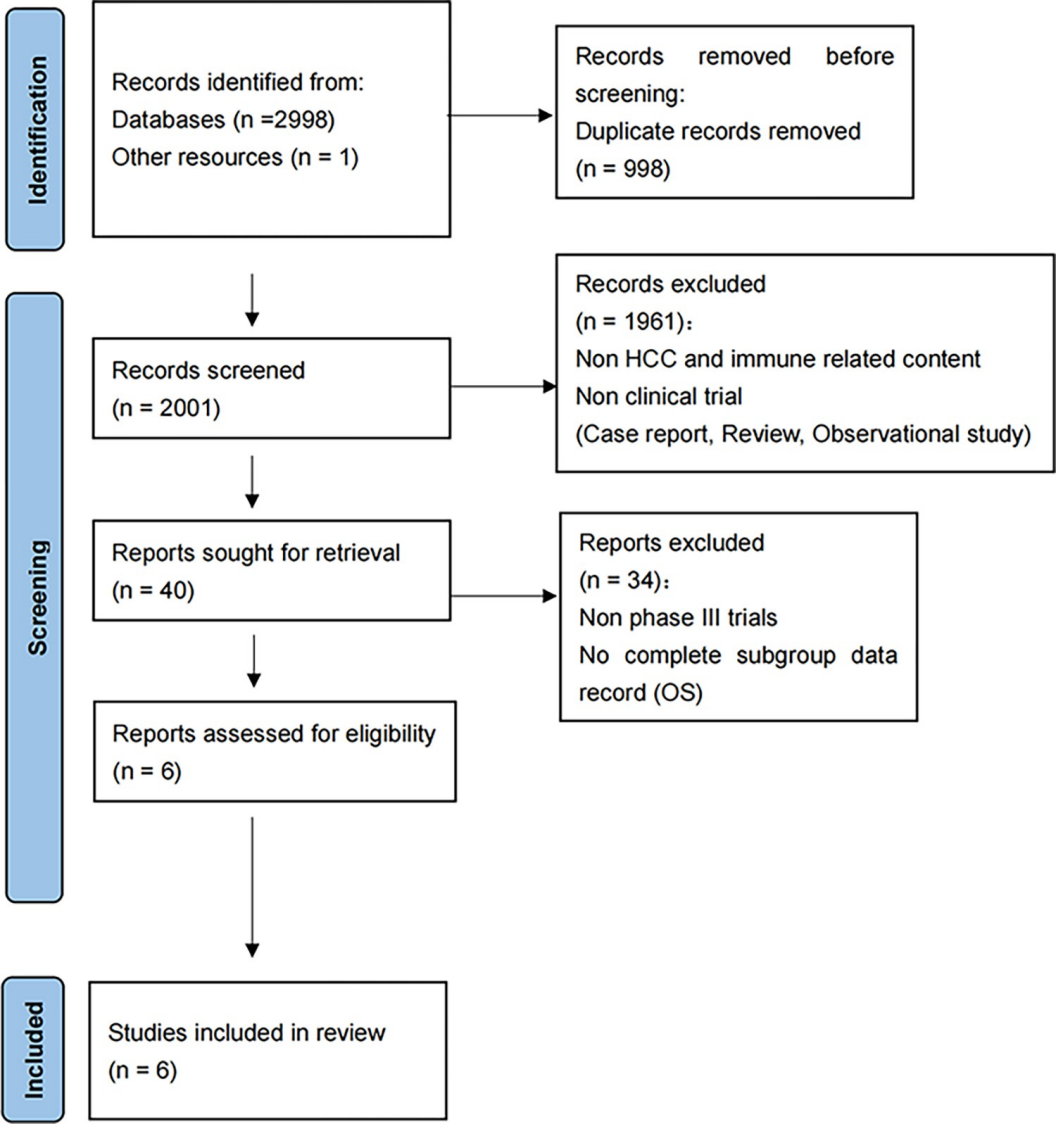
Flow diagram of study selection. HCC, hepatocellular carcinoma; OS, overall survival; PFS, progression-free survival; ORR, objective response rate.

The clinical trials included should conform to the below criteria: (1) **Study design**: studies should belong to first-line phase III randomized controlled trial (RCTs) in advanced HCC; (2) **Treatment:** the treatment arm of clinical trials should be immune combination therapies; (3) **Outcome**: hazard ratio (HR) values for endpoints OS were documented detailly in the subgroups of various clinico-characteristics. The clinical trials were excluded if they were (1) not in line with advanced HCC; (2) studies in which endpoints of interest (OS, PFS, or ORR) weren’t reported or no complete subgroup data record was provided; (3) not first-line phase III RCTs. The specific selection criteria and steps are shown in Fig 1.

### Quality assessment and data extraction

To evaluate potential bias of the included RCTs, the Cochrane Collaboration’s tool for assessing risk of bias [10] was utilized, which includes 6 fields and 7 evaluation items, resulting in studies being classified as “low bias,” “unclear,” or “high bias.” Details of the assessment of risk of bias can be found in the S1 Fig in S2 File.

The studies encompassed in this analysis underwent a detailed and comprehensive review, with keen attention paid to various intricacies including the identity of the primary author, the nature of the trial, the year of publication, the magnitude of the sample size, the median duration of follow-up, the specifics of the treatment and control arms, any adverse events recorded, and the primary endpoints established. The primary focus of the extraction process was on the HR of OS, with the pooled HR serving as the primary metric of interest. Secondary outcome measures included PFS, ORR, and toxicity profiles. The scoring and extraction tasks were carried out independently by TYC and LYR, and the results obtained were subsequently summarized by WYN.

### Statistical analysis

The studies encompassed within this analysis were systematically classified into three distinct categories, based on variations in the therapeutic approaches utilized: immunotherapy combined with anti-angiogenic agents, immunotherapy combined with TKIs, and dual immunotherapy [11].

The primary analysis data was extracted from the HR of OS and subsequently computed for each immune combination therapy to determine the corresponding values. Additionally, Bayesian random-effect network meta-analysis was conducted using R software (version 4.2.2, provided by The R Foundation) to ascertain the 95% credible interval (CrI). Moreover, the HR for PFS and the odds ratio for ORR were also comprehensively calculated. The statistical analysis of the different interventions in various clinico-characteristics was performed using the "gemtc" and "rjags" base packages. In addition, a Monte Carlo Markov Chain (MCMC) model and a consistency model were used to compare multiple treatments. 4 MCMC chains were conducted, the number of simulations reached 10000, and the number of iterations reached 50000. In order to evaluate convergence of the model, the track density plot and Brooks-Gelman-Rubin diagnostic diagram were used. If the density graph was normally distributed, the bandwidth tended to 0 and remained stable, the overlap region covered most of the chain fluctuation range, the convergence of the model was considered to be high. To assess the efficacy of various immune combination therapies, probability ranking diagrams were formulated utilizing the "Rank. Probability" functionality, along with the surface under the cumulative probability ranking (SUCRA). Additionally, the "anohe" function was employed to quantify the deviation of the I^2^ heterogeneity variance parameter. The incidences of adverse events were determined using both the arithmetic average method and risk ratio. To uphold the robustness of the analysis, a sensitivity analysis was executed via a case-by-case elimination approach.

## Results

### Characteristics of the included studies

Based on a rigorous search employing pre-established search terms, a comprehensive compilation of 2996 citations were retrieved, spanning the period from January 1, 2003 to December 31, 2023. Additionally, a single conference abstract published in 2022 was also included in this compilation. Subsequently, a diligent deduplication process led to the elimination of 998 redundant articles, resulting in a refined set of 2001 articles for further evaluation. These articles were carefully screened based on their titles and abstracts, ultimately identifying 40 studies that satisfied the initial selection criteria and were considered as potential candidates for further analysis. After a thorough assessment, six studies encompassing a total of 3840 patients were selected for inclusion in the meta-analysis. Notably, all of these studies were first-line phase III RCTs, ensuring the highest level of scientific rigor and reliability in our analysis.

The enclosed studies encompassed clinical trials pertaining to immunotherapy utilizing inhibitors of programmed death-(L)1. Within the corpus of studies encompassed in this analysis, two studies focused on immunotherapy in conjunction with anti-angiogenetic agents, specifically atezolizumab combined with bevacizumab and sintilimab coupled with bevacizumab. Three studies examined immunotherapy combined with TKIs, specifically atezolizumab plus cabozantinib [12], pembrolizumab plus lenvatinib, and camrelizumab combined with rivoceranib. The remaining study involved dual immunotherapy utilizing tremelimumab and durvalumab. Of the total 3840 subjects, 1986 participated in immunotherapy combined with TKIs, 1072 in immunotherapy with anti-angiogenetic agents, and 782 in dual immunotherapy. Among the six RCTs conducted for first-line treatment, four yielded positive outcomes, while two demonstrated negative results. Across all studies, the primary endpoints utilized were OS and PFS, with the exception of the HIMALAYA [7], which solely utilized OS as the primary endpoint. Additional details regarding the inclusion of studies are provided in Table 1.

**Table 1:** Characteristics of the studies included in network meta-analysis.

Trial	Group	n	Primary end point	Median follow-up (mo)	Phase	Treatment Arms	Control Arms	OS		PFS		0RR (%)
								Median (mo)	HR (95% CI)	Median (mo)	HR (95% CI)	
IMbrave-150 2021	Immunotherapy combined with anti-angiogenetic agents	501	OS PFS	17.6(0.1–28.6)/ 10.4(0–27.9)	Ⅲ	Atezolizumab combined with Bevacizumab	Sorafenib	19.2/13.4	0.66(0.52–0.85)	6.9/4.3	0.65(0.53–0.81)	30
ORIENT-32 2021	Immunotherapy combined with anti-angiogenetic agents	571	OS PFS	10(8.5–11.7)/ 10(8.4–11.7)	Ⅲ	Sintilimab combined with Bevacizumab	Sorafenib	NA/10.4	0.57(0.43–0.75)	4.6/2.8	0.56(0.46–0.70)	21
COSMIC-312 2022	Immunotherapy combined with tyrosine kinase inhibitors	649	OS PFS	13.3(10.5–16)	Ⅲ	Atezolizumab combined with Cabozantinib	Sorafenib	15.4/15.5	0.90(0.69–1.18)	6.8/4.2	0.63(0.44–0.91)	11
LEAP-002 2022	Immunotherapy combined with tyrosine kinase inhibitors	794	OS PFS	32.1	Ⅲ	Pembrolizumab combined with Lenvatinib	Lenvatinib+ Placebo	21.2/19.0	0.84(0.71–1.00)	8.2/8.0	0.87(0.73–1.02)	26.1
CARES-310 2023	Immunotherapy combined with tyrosine kinase inhibitors	543	OS PFS	NA	Ⅲ	Camrelizumab combined with Rivoceranib	Sorafenib	22.1/15.2	0.62(0.49–0.80)	5.6/3.7	0.52(0.41–0.65)	25.4
HIMALAYA 2022	Dual Immunotherapy	782	OS	33.18(31.74–34.53)/32.23(30.42–33.71)	Ⅲ	Tremelimumab combined with Durvalumab	Sorafenib	16.43/13.77	0.78(0.65–0.93)	3.78/4.07	0.90(0.77–1.05)	20.1

Immunotherapy specifically refers to the use of immune checkpoint inhibitors targeting PD-1, CTLA-4, and PD-L1. OS, overall survival; PFS, progression free survival; ORR, objective response rate; mo, month; HR, hazard ratio; CI, confidence interval; NA, not available.

### Analysis of immune combination therapies on advanced HCC patients: OS and subgroup evaluation based on diverse clinico-characteristics

**Fig 2:**
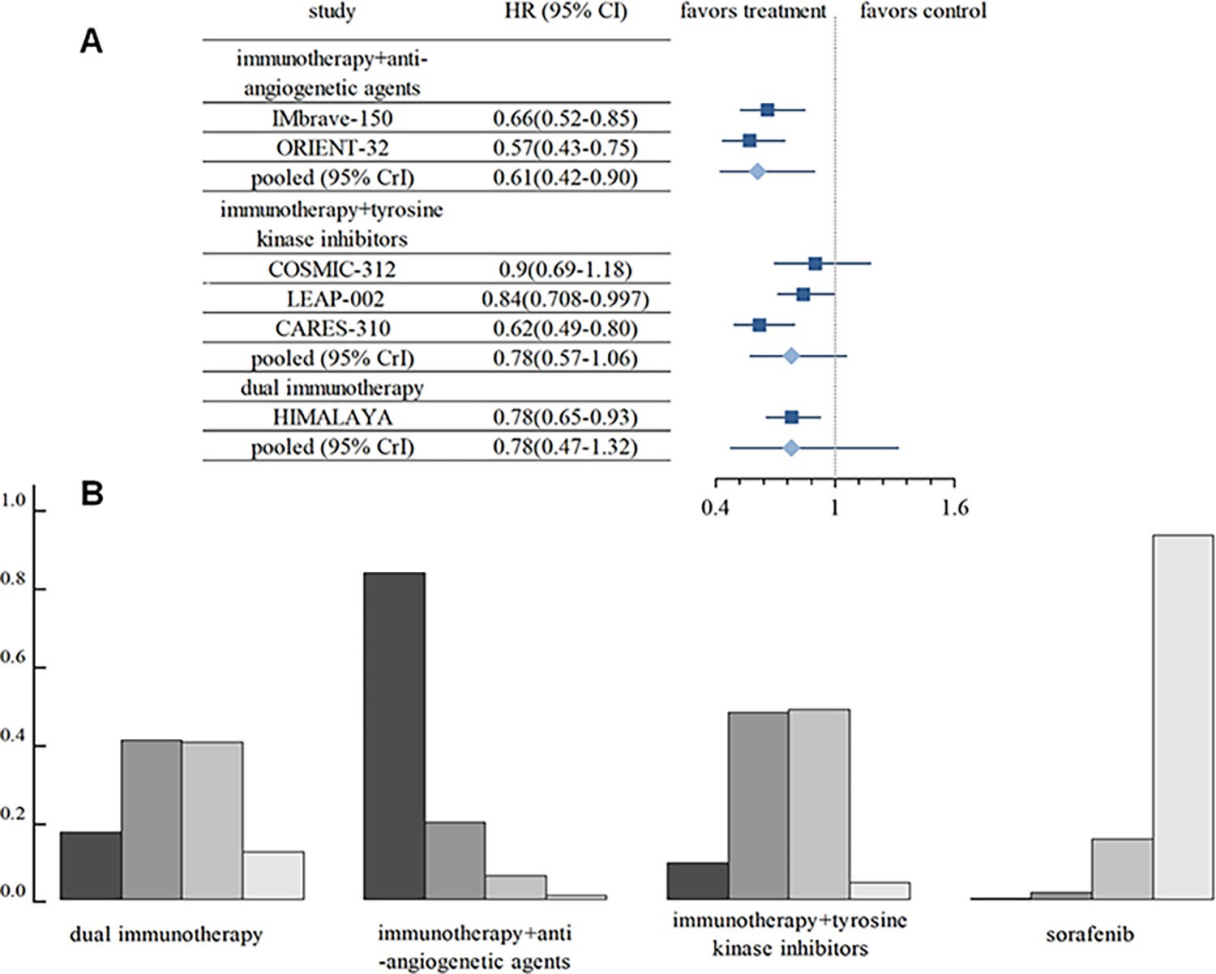
Comparison of overall survival among diverse immunotherapy regimens. (A) the forest plot for the outcome of overall survival (OS) using hazard ratio (HR) values and corresponding 95% credible interval (CrIs), (B) The ranking probability histogram for the outcome of OS.

A total of six studies, encompassing the three interventional arms, were subjected to rigorous analysis regarding the endpoint of overall survival (OS). The pooled HR and its corresponding 95% CrI for the comparisons among these treatment modalities were presented in Fig 2. Specifically, Fig 2A illustrates that the integration of immunotherapy with anti-angiogenetic agents led to significant improvements in OS compared to sorafenib (HR = 0.61, 95% CrI, 0.42–0.90). It is noteworthy, however, that no statistically significant differences were observed in the OS hazard ratios among the three immune combination therapy strategies, as detailed in S2A Table in S2 File. To further elucidate the relative efficacies of these therapies, a histogram depicting the ranking probabilities was constructed. This analysis clearly demonstrates that, among the four interventional approaches (depicted in Fig 2B), the combination of immunotherapy with anti-angiogenetic agents emerged as the most promising strategy, whereas sorafenib exhibited the highest likelihood of being the least effective. Consequently, it can be logically inferred that the descending order of benefit in terms of OS is as follows: immunotherapy combined with anti-angiogenetic agents, immunotherapy combined with TKIs, dual immunotherapy, and sorafenib.

**Fig 3:**
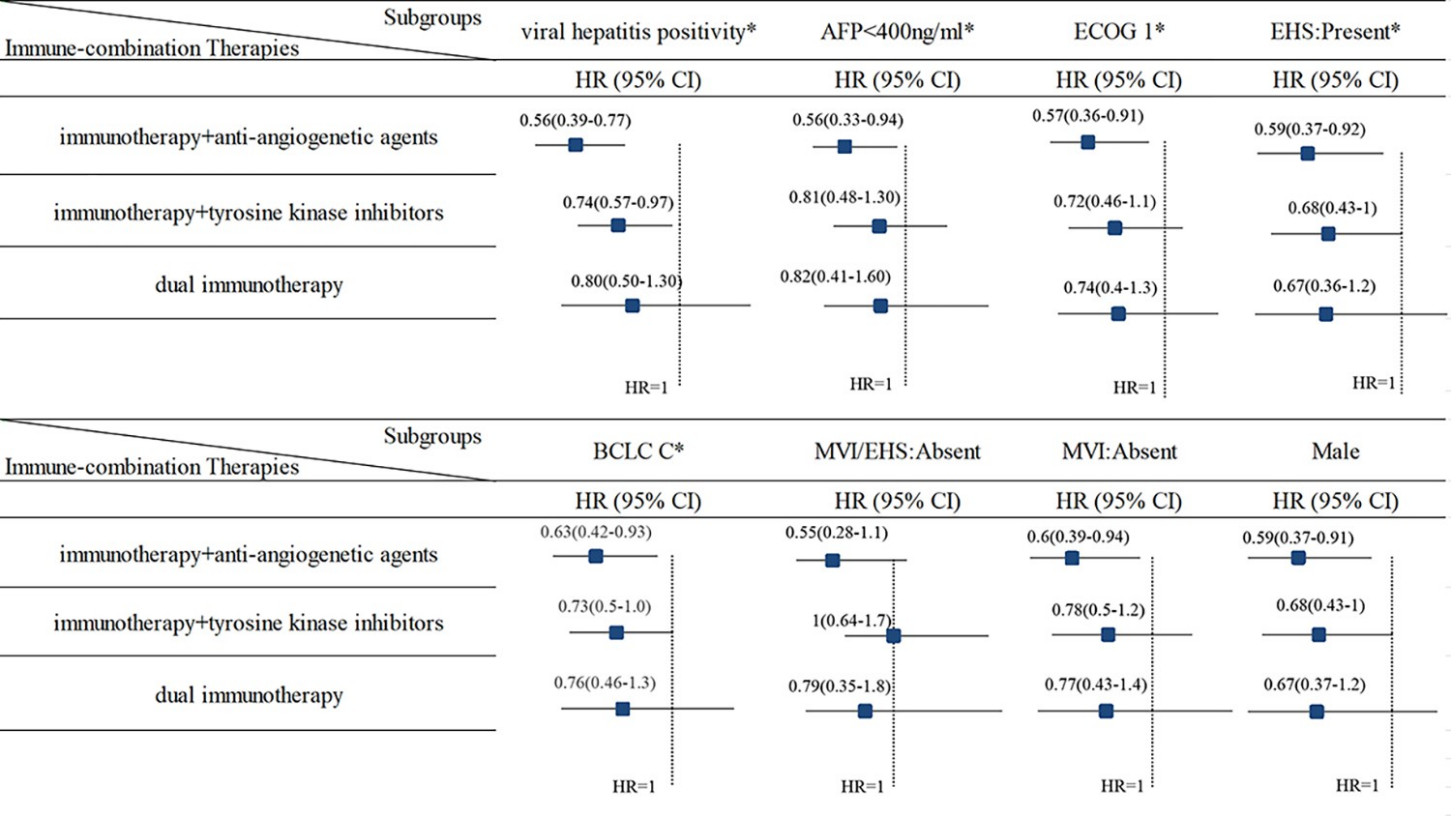
The subgroup analysis of diverse clinico-characteristics. The results of subgroups were all utilizing the sorafenib/lenvatinib as control group. * Represents to the clinico-characteristics has difference among three immune combination therapies and stays stable after removing the LEAP-002 study. AFP, alpha-fetoprotein; BCLC C, Barcelona Clinic Liver Cancer C; ECOG 1, Eastern Cooperative Oncology Group 1; EHS, extrahepatic spread; MVI, macrovascular invasion.

A subgroup analysis was conducted to assess the impact of various clinico-characteristics on the efficacy of immune-combination therapies. A comprehensive evaluation of six studies, encompassing a total of 3840 patients, was performed to report on OS outcomes across diverse clinico-pathological profiles. The detailed findings and results are graphically presented in Fig 3, leading to the conclusion that immunotherapy combined with anti-angiogenetic agents demonstrated superior therapeutic efficacy in patients characterized by positive viral hepatitis status (HR = 0.56, 95% CrI, 0.39–0.77), AFP levels<400 ng/ml (HR = 0.56, 95% CrI, 0.33–0.94), ECOG 1 (HR = 0.57, 95% CrI, 0.36–0.91), presence of extrahepatic spread (EHS) (HR = 0.59, 95% CrI, 0.37–0.92), and BCLC C (HR = 0.63, 95% CrI, 0.42–0.93).

**Fig 4:**
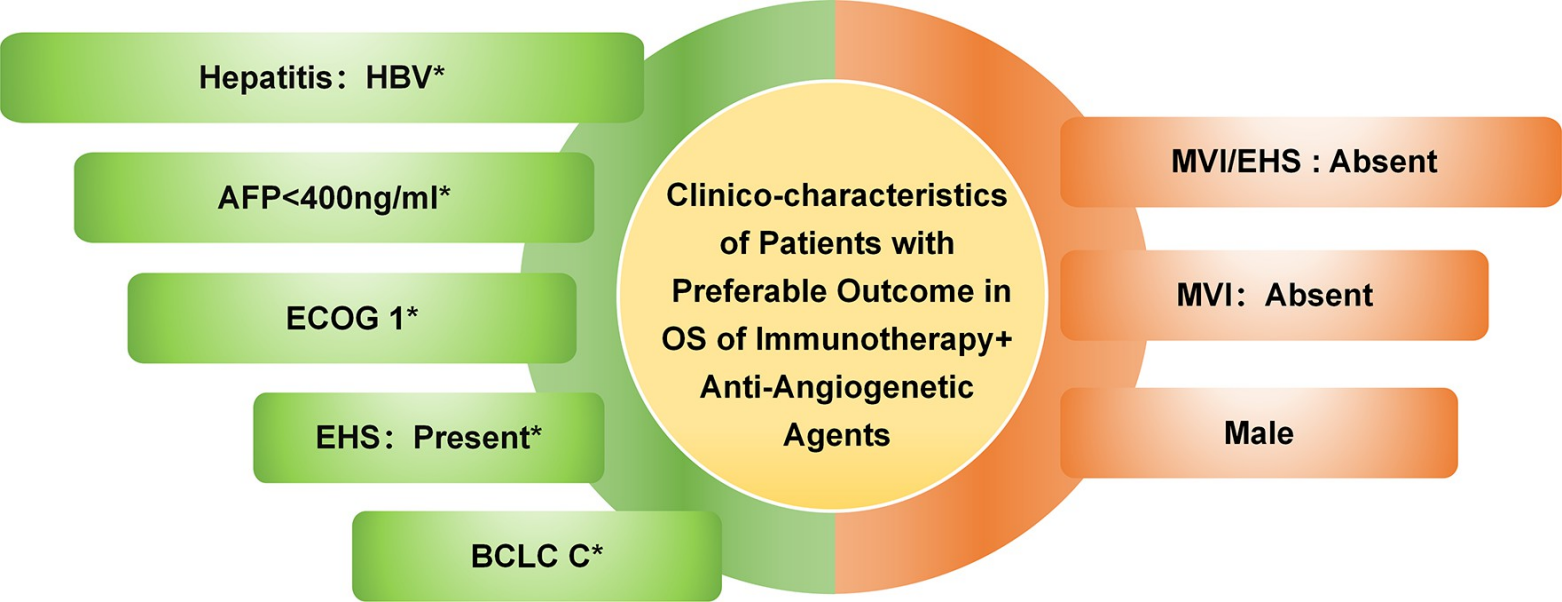
Clinico-characteristics of patients with preferable outcome in overall survival of immunotherapy combined with anti-angiogenetic agents. Clinico-pathological characteristics with * were that characteristics had preferable response in OS when using immunotherapy combined with anti-angiogenetic agents. AFP, alpha-fetoprotein; BCLC C, Barcelona Clinic Liver Cancer C; ECOG 1, Eastern Cooperative Oncology Group 1; EHS, extrahepatic spread; MVI, macrovascular invasion.

Furthermore, the overall trend in the ranking of efficacy was consistent with previously reported findings, indicating that immunotherapy combined with anti-angiogenetic agents was more effective than immunotherapy combined with TKIs, dual immunotherapy, and sorafenib. The clinico-characteristics associated with favorable OS outcomes in patients receiving immunotherapy combined with anti-angiogenetic agents are summarized in Fig 4. Notably, the observed trend was particularly pronounced among the subgroup of patients with positive viral hepatitis. To further refine the evaluation of efficacy among patients with positive viral hepatitis, separate comparisons were conducted between patients with positive HBV or hepatitis C virus (HCV) and those without hepatitis. Interestingly, the order of therapeutic benefit remained unchanged, particularly within the HBV subgroup, as illustrated in S2 Fig in S2 File.

To guarantee the robustness of the study findings, LEAP-002, utilizing lenvatinib as the control arm, was omitted from subsequent analytical considerations. Notably, the ensuing analysis produced concordant results that were in harmony with our previously drawn conclusions, as exhibited in S3 Fig in S2 File.

### PFS and ORR analysis of immune combination therapies on advanced HCC patients

With regard to the endpoint of PFS, as depicted in S4A Fig in S2 File, the combination of immunotherapy and anti-angiogenetic agents appears to marginally enhance PFS compared to sorafenib (HR = 0.60, 95% CrI, 0.36–0.99. To further strengthen the robustness and credibility of our findings, a reassessment was undertaken excluding the LEAP-002 data. Subsequently, the analysis revealed that immunotherapy combined with TKIs exhibited the most pronounced beneficial effect, closely followed by immunotherapy in conjunction with anti-angiogenetic agents, as illustrated in S4B Fig in S2 File. Based on these observations, it is reasonable to conclude that immunotherapy, when combined with anti-angiogenetic agents, demonstrates favorable efficacy in terms of PFS.

As illustrated in S4A Fig in S2 File, immunotherapy combined with anti-angiogenetic agents and immunotherapy combined with TKIs exhibited a superior ORR compared to sorafenib, (OR = 4.49, 95% CrI, 1.23–17.7; OR = 2.99, 95% CrI, 1.04–9.06, respectively). According to the ranking probability histogram, dual immunotherapy emerged as a relatively more effective therapeutic option, closely followed by immunotherapy combined with anti-angiogenetic agents. Consequently, it can be deduced that immunotherapy combined with anti-angiogenetic agents occupies a favorable position in terms of the observed benefit in ORR.

### Convergence, inconsistency and sensitivity analysis

The NMA conducted a comprehensive comparison of the therapeutic efficacies associated with various immune-combination strategies. These strategies encompassed immunotherapy paired with anti-angiogenetic agents, immunotherapy in conjunction with TKIs, and dual immunotherapy. Each treatment arm was graphically represented by circles, with the relative thickness of the corresponding lines serving as a proportional indicator of the number of studies incorporated into the respective intervention (as depicted in S5 Fig in S2 File). To assess the convergence of the models, a range of analytical tools were employed, including trace and density plots, as well as the Brooks-Gelman-Rubin diagnostic diagram. Visual inspection revealed that the single-chain fluctuations were not discernible, and the density plots exhibited a normal distribution, both indicating satisfactory convergence of the models (as presented in S6 Fig in S2 File). Heterogeneity analysis revealed the presence of heterogeneity in the comparison specifically between immunotherapy combined with TKIs and sorafenib (as shown in S7 Fig in S2 File). However, it is noteworthy that no heterogeneity was observed in the overall combined analysis. To further validate the robustness of the findings, a sensitivity analysis was conducted utilizing the one-by-one elimination. This approach ensured the stability of the analysis results, thus enhancing the reliability of the conclusions drawn (as illustrated in S8 Fig in S2 File).

### Toxicity and specific treatment-related adverse events

**Fig 5:**
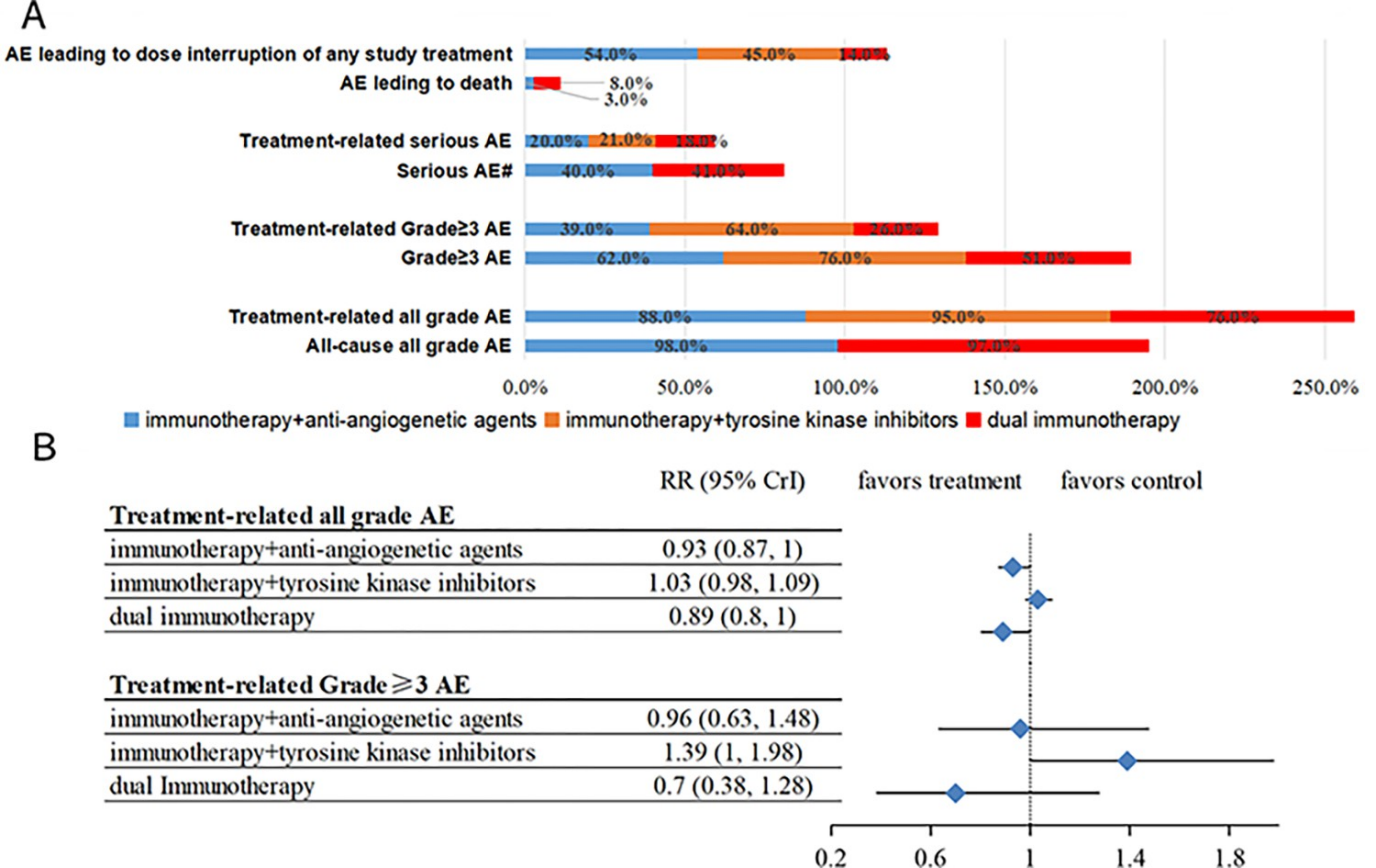
Comparison of toxicity among diverse immunotherapy regimens. (A) bar chart-comparison of adverse events among diverse immunotherapy regimens, (B) the Forest plot-comparison of treatment-related adverse events among diverse immunotherapy regimens. AE, adverse event; RR, risk ratio.

When assessing the utilization of a drug, it is imperative to meticulously consider its effectiveness and safety. Furthermore, the studies included in our analysis were systematically categorized into three distinct groups based on the treatment protocol employed. The toxicity associated with each group was subsequently quantified using arithmetic averaging techniques and risk ratio calculations. As depicted in Fig 5, the occurrence of TRAEs of all grades and grade≥3 AE appears to be lower in patients undergoing dual immunotherapy, in comparison to those treated with immunotherapy combined with TKIs, which exhibited the highest frequency of such adverse events. In summary, our findings reveal that the overall toxicity prevalence was lowest in the cohort receiving dual immunotherapy, followed by those treated with immunotherapy combined with anti-angiogenetic agents, and lastly, immunotherapy combined with TKIs.

The rates of frequently occurring specific TRAEs are summarized in S3 Table in S2 File. Regarding immunotherapy combined with anti-angiogenetic agents, among the fourteen enumerated TRAEs, proteinuria (36%), hypertension (30%), and bilirubin elevation (29%) emerged as the three most prevalent adverse effects. For immunotherapy in conjunction with TKIs, diarrhea (48%), hypertension (41%), and proteinuria (36%) were the most frequently reported. Finally, in the context of dual immunotherapy, diarrhea (27%), pruritus (23%), and rash (22%) were the top three adverse events observed.

## Discussion

This study presented a systematic review and network meta-analysis of immune-combination therapy trials conducted for patients with advanced HCC, spanning from January 2003 to December 2023. Following a rigorous quality evaluation of 2999 pertinent articles, six studies encompassing a total of 3840 patients were selected for inclusion. The study further elucidates the efficacy of three first-line immune-combination therapies in terms of OS among patients with advanced HCC, stratified into various subgroups based on their clinicopathological characteristics.

Currently, observational studies are insufficient for comparing multiple interventions. NMA addresses this limitation by allowing for direct and indirect comparisons that rank the efficacy of various treatments. This study serves as a groundbreaking effort in utilizing NMA to evaluate the effectiveness of immunotherapy combined with anti-angiogenetic agents, immunotherapy combined with TKIs, and dual immunotherapy for patients with advanced HCC, across diverse subgroups defined by their clinico-characteristics.

An overall comparison of efficacy revealed that immunotherapy combined with anti-angiogenetic agents may exert the most substantial therapeutic impact, thereby resulting in substantial enhancements in OS. The findings of this study align with those reported in previous NMA studies, which have shown a pronounced superiority of the combined atezolizumab and bevacizumab regimen in first-line treatment [13, 14].

Despite the overall favorable prognosis of immunotherapy for advanced HCC, it is important to acknowledge that not all patients derive effective treatment outcomes [15]. Moreover, a substantial proportion of treated patients, up to a quarter, encounter significant immune-related adverse events [16]. Therefore, the identification of the dominant population or predictive advantaged clinico-characteristics for immunotherapy remains ongoing research. A subgroup analysis of the data has revealed promising efficacy in terms of OS among patients with Barcelona Clinic Liver Cancer (BCLC) C, Eastern Cooperative Oncology Group (ECOG) 1, presence of EHS, AFP levels<400 ng/ml, and viral hepatitis positivity, particularly HBV, when treated with immunotherapy combined with anti-angiogenetic agents. In comparison to other immune combination therapies, the atezolizumab-bevacizumab and sintilimab-bevacizumab regimens have demonstrated superior OS outcomes in patients with BCLC C, with HR of 0.63 for both regimens. Consequently, the atezolizumab-bevacizumab regimen has emerged as the preferred systemic therapy option for these patients [17]. Similarly, patients with ECOG 1 treated with immunotherapy combined with anti-angiogenetic agents have also demonstrated favorable survival benefits (HR = 0.56 and 0.59, respectively). While initial concerns regarding the OS response of patients with EHS to immune checkpoint inhibitors (ICIs) persisted [18], recent phase III clinical studies have consistently shown improved OS outcomes in this patient population [3, 19]. Furthermore, a comparative analysis of ICIs treatments has revealed that patients with EHS exhibit preferential OS responses when treated with immunotherapy combined with anti-angiogenetic agents. Additionally, studies have indicated that patients with AFP levels < 400 ng/ml may benefit from immunotherapy [20]. This advantage may be attributed to the association between low AFP levels and smaller tumor size, improved liver function, and reduced recurrence rates [21, 22]. Notably, the FDA has approved the use of Ramucirumab as a monotherapy in patients with AFP levels of 400 ng/mL or higher following sorafenib treatment. Consequently, further research is necessary to clarify the relationship between AFP levels and immune benefits in advanced liver cancer.

The results of this NMA revealed that patients with positive viral hepatitis positivity who undergo a combined treatment of immunotherapy and anti-angiogenic agents exhibit significantly positive outcomes. A rigorous meta-analysis has concurred with these findings, indicating that viral hepatitis responds more favorably to immunotherapy compared to non-viral hepatitis. This may be attributed to the enhanced immunogenicity of novel peptides derived from viruses [23, 24]. The accumulating evidence from various research studies clearly demonstrates that non-viral HCC, particularly HCC associated with non-alcoholic steatohepatitis, exhibits a reduced response to the CD8 combined with PD1 immune checkpoint inhibitors, along with T cell self-invasion behavior, in comparison to virus-associated HCC [25, 26]. These observations strongly indicate that viral hepatitis exhibits a more beneficial response to ICIs.

Anti-angiogenic agents are highly regarded as an effective method for enhancing anti-tumor immunity in cancer patients, especially when used concurrently with immunotherapy [27]. When combined with anti-PD-(L)1 agents, these drugs demonstrate a synergistic potential that augments anti-tumor therapy [28]. Specifically, anti-angiogenic medications optimize the tumor microenvironment, mitigate the suppressive effects of the tumor on immune cells, and enhance the infiltration and functionality of immune cells, thereby intensifying the immunotherapeutic effect of PD-(L)1 inhibitors. Additionally, these agents impede tumor angiogenesis and restrict the tumor’s nutrient supply, rendering it more susceptible to immune recognition and attack [4, 27, 29, 30]. This synergistic action may primarily account for the superior efficacy observed in OS when immunotherapy is combined with anti-angiogenic agents.

In the context of HCC, several TKIs are commonly utilized, including cabozantinib [31], Lenvatinib [32], and rivoceranib [33, 34]. The therapeutic efficacy of combining TKIs with ICIs is primarily attributed to the suppression of VEGF signaling. The concerted action of these two agents effectively inhibits tumor-associated macrophages and regulatory T cells [35], thereby mitigating their suppressive effects on the immune microenvironment. Consequently, there is a decrease in the levels of TGF-β and IL-10, downregulation of PD-1 and T cell immunoglobulin and mucin domain 3 expression, and an enhancement of the immunostimulatory cytokine IL-12, ultimately fostering an immune response conducive to antitumor immunity [36, 37]. However, unlike anti-angiogenic agents, which possess long half-lives and exhibit high specificity through the inhibition of protein interactions, TKIs typically exhibit shorter half-lives and enter cells through traditional chemical drug mechanisms to target intracellular molecules. Therefore, the synergistic potential of anti-angiogenic agents combined with anti-PD-(L)1 drugs is generally considered to be superior to the combination of TKIs.

Dual immunotherapy for advanced HCC leverages the synergistic action of cytotoxic T lymphocyte-associated antigen-4 and PD-L1 monoclonal antibodies to activate T cells, resulting in their rapid activation and extensive proliferation [38]. During the activation stage of T cells, the concurrent administration of CTLA-4 and PD-L1 inhibitors synergistically enhances the continuous and accelerated activation of effector T cells, facilitating their extensive proliferation and migration towards the tumor microenvironment [39]. Subsequently, in the effector phase, the combined effect of CTLA-4 and PD-L1 inhibitors effectively overcomes the immunosuppressive “brake” exerted by tumor cells, thereby attaining a high level of activation and rapid elimination of tumor cells [40, 41]. HIMALAYA, currently the sole approved first-line dual immunotherapy for advanced HCC, has demonstrated significant improvements in patient survival by harnessing the endogenous immune capacity of patients, offering a renewed ray of hope for those suffering from advanced HCC [7, 42]. Although its impact on overall survival may not be as profound as immunotherapy combined with anti-angiogenic agents, the comparative outcomes with immunotherapy combined with TKIs remain inconclusive and require further exploration in future research endeavors.

In clinical drug decision-making, both the efficacy and toxicity of the treatment regimen constitute crucial considerations. This study has demonstrated that dual immunotherapy exhibits minimal toxicity, likely attributable to durvalumab’s (PD-L1) capability of preserving immune homeostasis within normal tissues, minimizing autoimmune response in those tissues, and mitigating the risk of toxicity [43]. Analogously, atezolizumab performs a comparable function when combined with anti-angiogenic agents, thereby reducing the toxicity associated with bevacizumab and resulting in a lower toxicity profile compared to immunotherapy combined with TKIs.

The study also had certain limitations. Firstly, the paucity of pertinent publications necessitates the absence of a closed loop in the NMA, thus impeding the comprehensive analysis of the consistency of comparative outcomes. Nevertheless, the NMA remains instrumental in establishing a ranking of potential efficacy and safety profiles, thereby facilitating clinical decision-making. Secondly, the LEAP-002 study utilized lenvatinib as the control group, which was deemed equivalent to sorafenib in the present study. An earlier randomized phase III study had established the comparability of lenvatinib and sorafenib in terms of their therapeutic efficacy, with lenvatinib demonstrating non-inferiority to sorafenib in efficacy [44]. Additionally, we conducted a reanalysis excluding the research findings of LEAP-002, and the resultant outcomes were largely concordant with the current study’s findings, thereby preserving the validity of our conclusions.

Given the rapid pace of development in immunotherapy, a series of first-line phase III RCTs that conducted on immune combination therapies for advanced HCC are in progress. Furthermore, the results of this study could serve as a valuable clinical reference, assisting physicians in selecting the most appropriate and optimal treatment options and aiding in decision-making.

## Conclusion

In summary, immunotherapy combined with anti-angiogenetic agents holds promise as a superior therapeutic strategy in patients diagnosed with BCLC C stage, ECOG 1, EHS, AFP levels<400ng/ml, and viral hepatitis positivity, particularly HBV. Of the three immune-combination therapy regimens, this specific approach could be regarded as the most effective first-line treatment option for patients with advanced HCC, especially those with viral hepatitis positivity, in terms of OS, when contrasted with sorafenib. Additionally, the toxicity associated with immunotherapy combined with anti-angiogenetic agents is considered moderate.

## Supporting information

S1 File PRISMA 2020 checklist. (DOCX)

S2 File Supplementary material. (PDF)

## Abbreviations

AFP: alpha fetoprotein
CrI: credible interval
CTLA-4: cytotoxic T lymphocyte-associated antigen-4
ESMO: European society for medical oncology
HBV: hepatitis B virus
HCC: hepatocellular carcinoma
HR: hazard ratio
LT: liver transplantation
MCMC: Monte Carlo Markov Chain
NMA: network meta-analysis
OS: overall survival
PD-L1: programmed cell death-Ligand 1
PFS: progression-free survival
RCT: randomized controlled trial
SUCRA: surface under the cumulative probability ranking
TRAE: treatment-related adverse event
VEGF: vascular endothelial growth factor
