# Supplementary material for: Efficacy comparison of immune combination therapies in subgroups for advanced hepatocellular carcinoma patients: Systematic review and network meta-analysis
Source: PLoS One. 2024 Jul 22;19(7):e0306869. doi: 10.1371/journal.pone.0306869 (PMC11262675; doi:10.1371/journal.pone.0306869)
Supplement: S2 File — (PDF) [file pone.0306869.s002.pdf]

Efficacy comparison of immune combination therapies in subgroups for advanced hepatocellular carcinoma patients: systematic review and network meta-analysis

Supporting Information

- S1 Fig. Risk assessment of bias.
- S2 Fig. Comparison of efficacy in OS between subgroups of hepatitis B virus and hepatitis C virus.
- S3 Fig. The subgroup analysis of diverse clinico-characteristics after removing the LEAP-002 study
- S4 Fig. Comparison of progression-free survival and objective response rate among diverse immunotherapy regimens.
- S5 Fig. Network diagram.
- S6 Fig. Trace and density plot, and Brooks-Gelman-Rubin diagnostic plot for fit degree test.
- S7 Fig. Heterogeneity test of overall survival, progression-free survival and objective response rate.
- S8 Fig. Sensitivity analysis of overall survival, progression-free survival and objective response rate.
- S1 Table. Detailed search strategy.
- S2 Table. The commutatively comparative efficacy of different immune combination therapies.
- S3 Table. Specific treatment-related adverse events of the immune combination therapies.

S1 Fig. Risk assessment of bias

a. Risk of bias summary

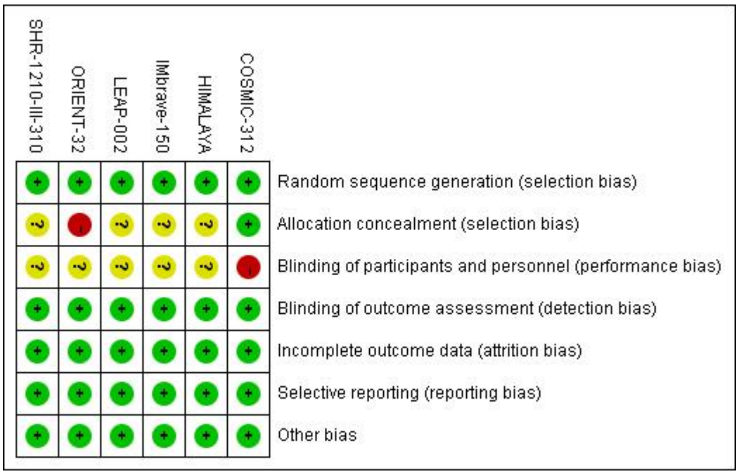

b. Risk of bias graph

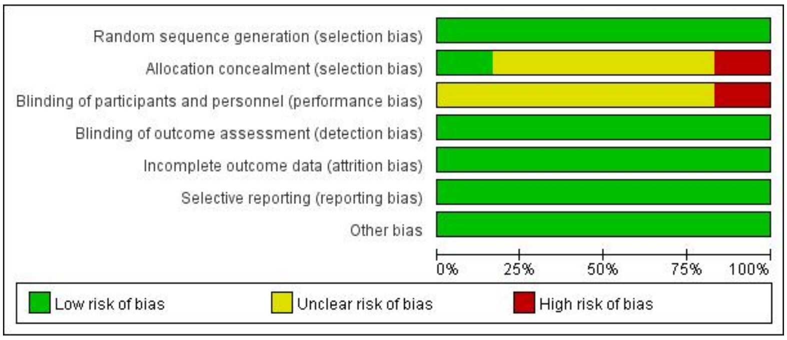

S2 Fig. Comparison of efficacy in OS between subgroups of hepatitis B virus and hepatitis C virus

a.

Viral Hepatitis

**HBV**	HR (95% CrI)
immunotherapy+anti-angiogenetic agents	0.58 (0.40, 0.85)
immunotherapy+tyrosine kinase inhibitors	0.67 (0.48, 0.90)
dual immunotherapy	0.64 (0.38, 1.1)
**HCV**	
immunotherapy+anti-angiogenetic agents	0.43 (0.15, 1.2)
immunotherapy+tyrosine kinase inhibitors	0.86 (0.44, 1.5)
dual Immunotherapy	1.1 (0.41, 2.8)

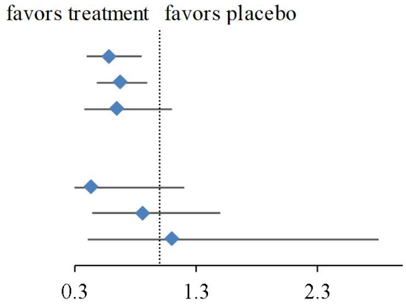

b.

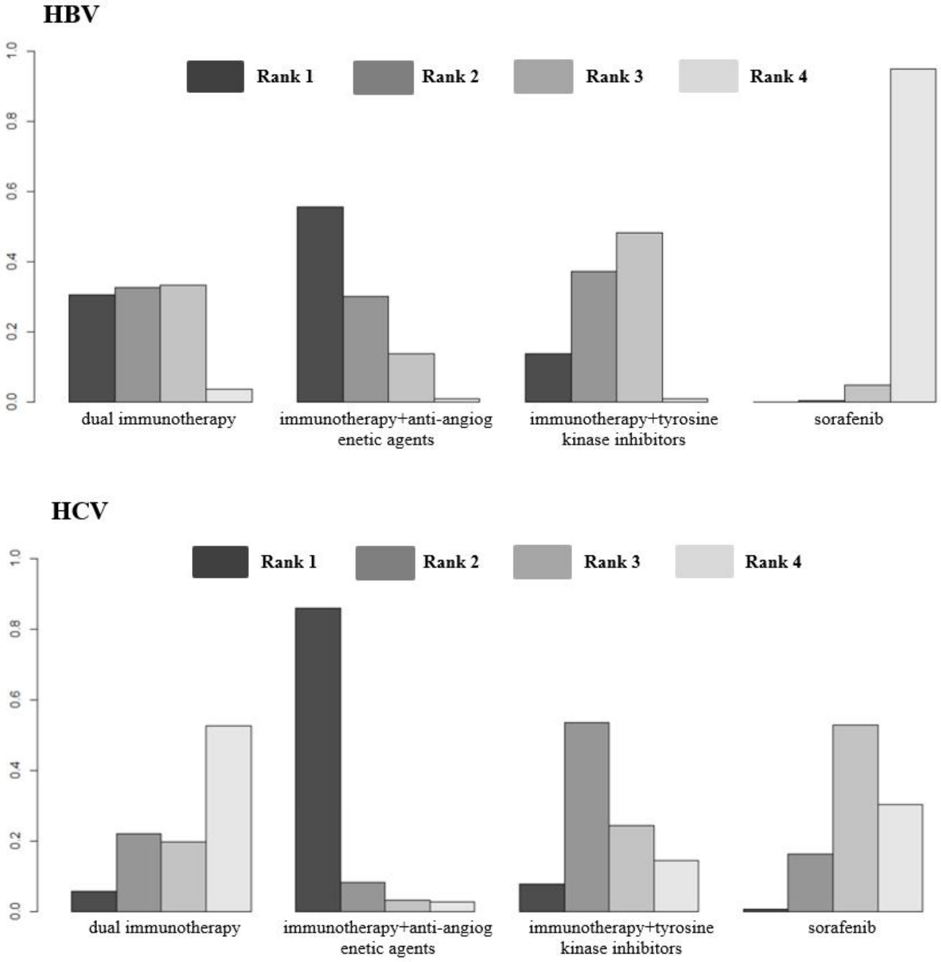

S2 Fig. (a) The forest plot for the outcome of overall survival (OS) using hazard ratio values and corresponding 95% credible interval. (b)The ranking probability histogram for the outcome of OS.

S3 Fig. The subgroup analysis of diverse clinico-characteristics after removing the LEAP-002 study.

Outcome measures

overall survival

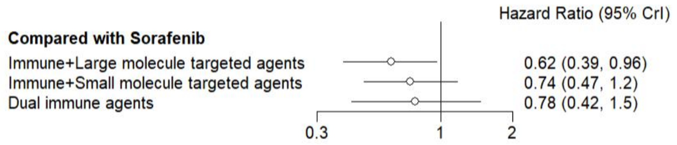

progression-free survival

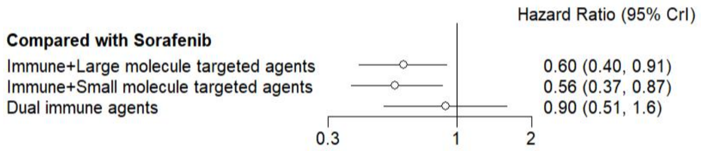

objective response rate

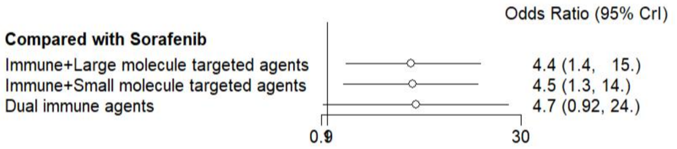

Clinico-characteristics

viral hepatitis positivity

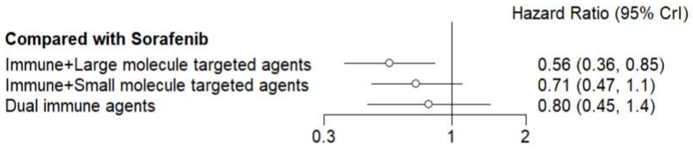

AFP<400ng/ml

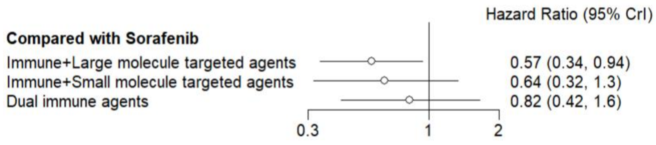

EHS: present

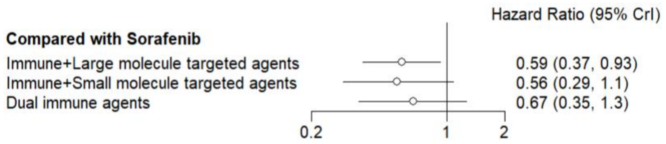

BCLC C

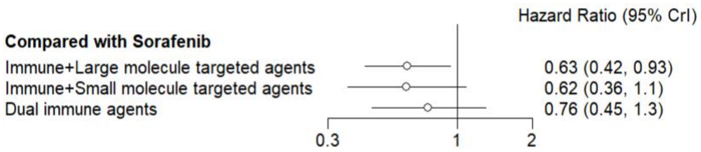

ECOG 1

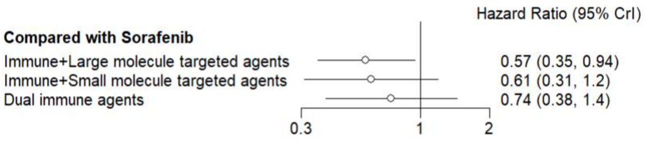

MVI/EHS: Absent

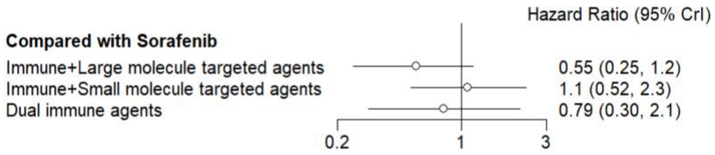

MVI: Absent

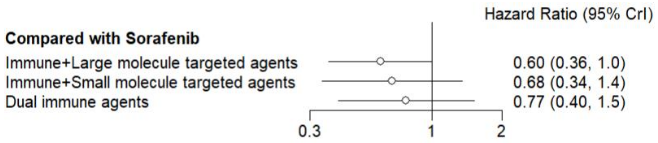

S3 Fig. AFP, alpha-fetoprotein; BCLC C, Barcelona Clinic Liver Cancer C; ECOG 1, Eastern Cooperative Oncology Group 1; EHS, extrahepatic spread; MVI, macrovascular invasion. Immune+Large molecule targeted agents refers to immunotherapy+anti-angiogenetic agents; Immune+Small molecule targeted agents refers to immunotherapy+tyrosine kinase inhibitors.

S4 Fig. Comparison of progression-free survival and objective response rate among diverse immunotherapy regimens

a. forest plots

PFS

study	HR (95% CrI)
immunotherapy+anti-angiogenetic agents	0.60 (0.36, 0.99)
immunotherapy+tyrosine kinase inhibitors	0.67 (0.44-1.01)
dual immunotherapy	0.90 (0.45-1.78)

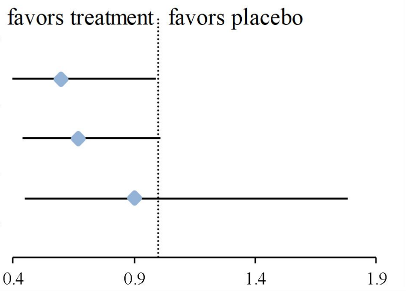

ORR

study	0R (95% CrI)
immunotherapy+anti-angiogenetic agents	4.49 (1.23, 17.7)
immunotherapy+tyrosine kinase inhibitors	2.99 (1.04, 9.06)
dual immunotherapy	4.72 (0.74, 30.11)

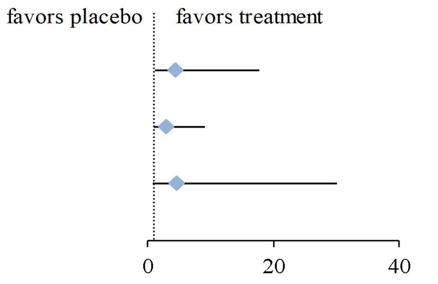

b. the ranking probability histograms

PFS

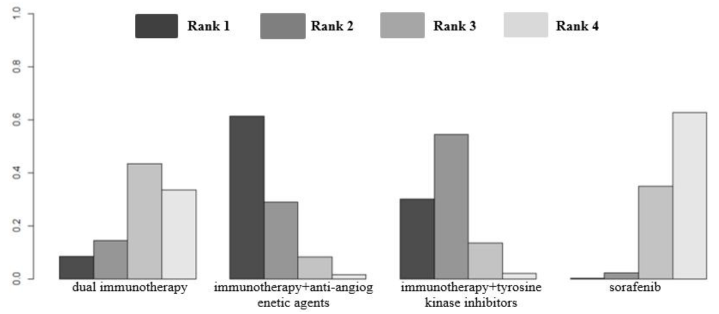

ORR

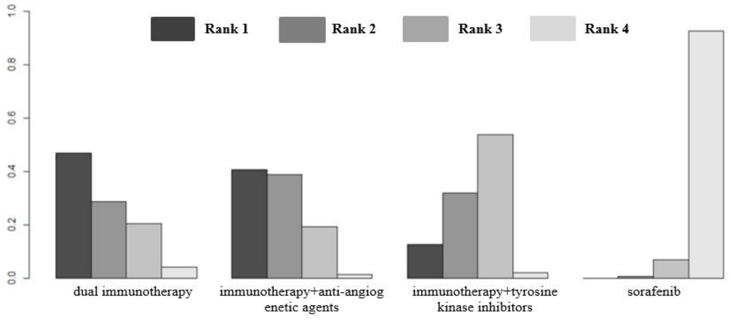

S4 Fig. (a) The forest plot for the outcome of objective response rate using odds ratio values and corresponding 95% credible interval. (b)The ranking probability histogram for the outcome of ORR.

S5 Fig. Network diagram

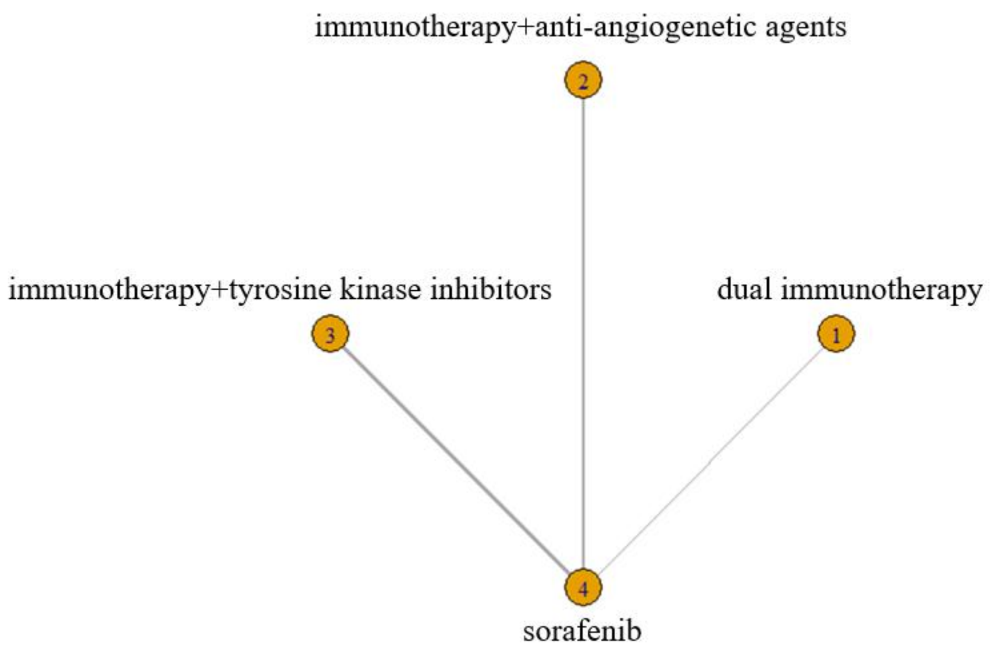

S6 Fig. Trace and density plot, and Brooks-Gelman-Rubin diagnostic plot for fit degree test

a. Overall survival

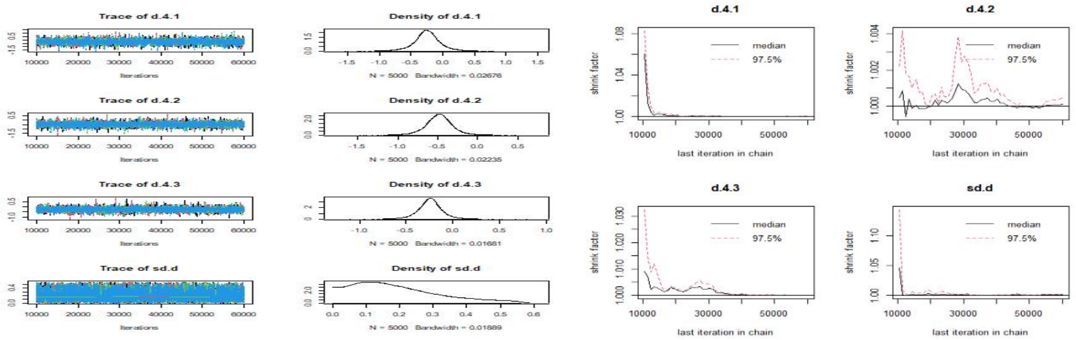

b. Progression-free survival

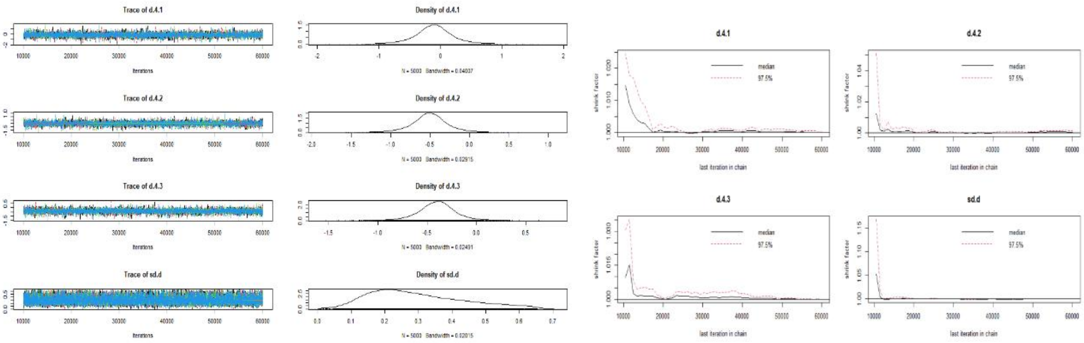

c. Objective response rate

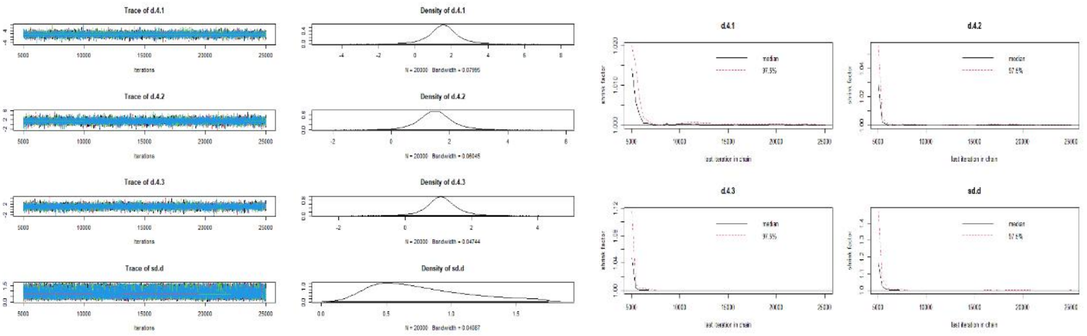

S6 Fig. 1-dual immunotherapy; 2-immunotherapy+anti-angiogenetic agents; 3-immunotherapy+tyrosine kinase inhibitors; 4-sorafenib.

S7 Fig. Heterogeneity test of overall survival, progression-free survival and objective response rate

a. overall survival

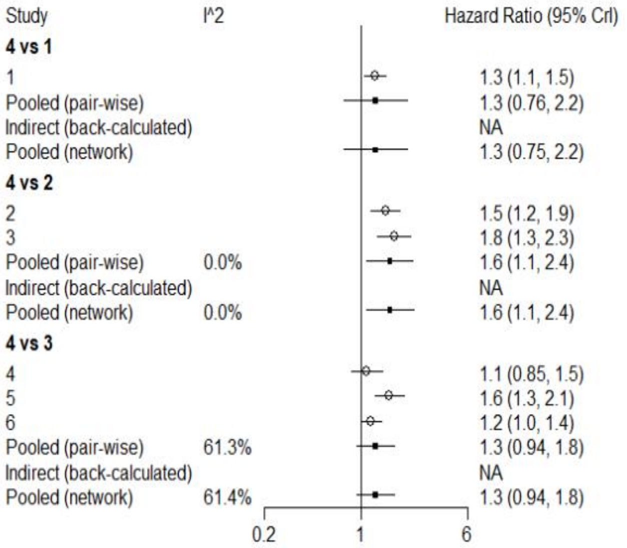

b. progression-free survival

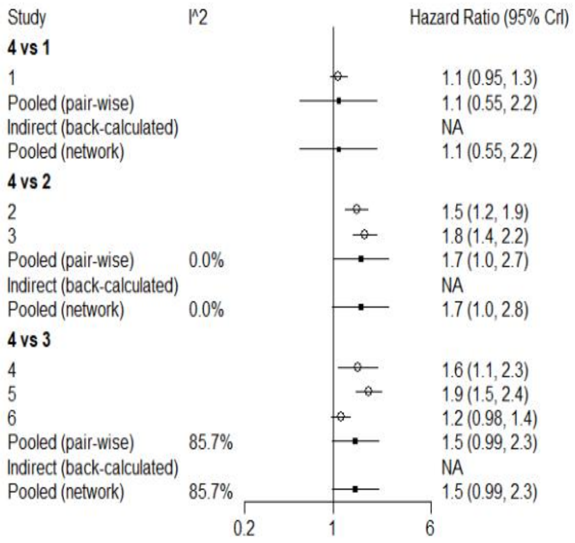

c. objective response rate

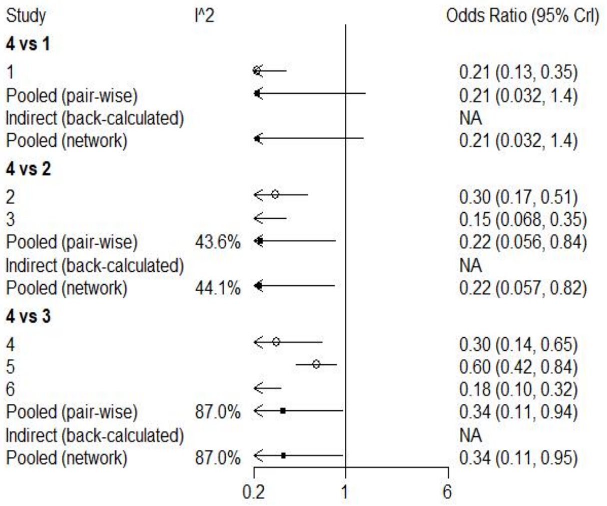

S7 Fig. 1-dual immunotherapy; 2-immunotherapy+anti-angiogenetic agents; 3-immunotherapy+tyrosine kinase inhibitors; 4-sorafenib.

S8 Fig. Sensitivity analysis of overall survival, progression-free survival and objective response rate

a. overall survival

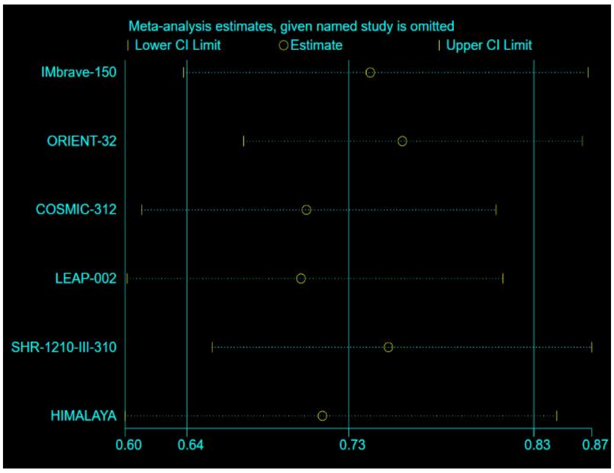

b. progression-free survival

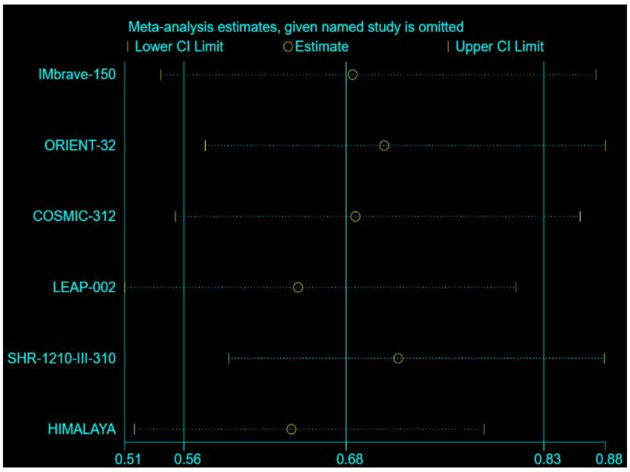

c. objective response rate

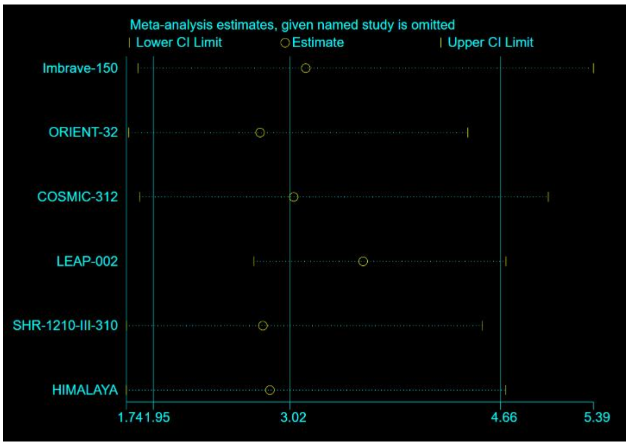

S1 Table. Detailed search strategy

Search	Database	Query	Results
**1**	PubMed	(((("carcinoma, hepatocellular"[MeSH Terms] OR "hepatocellular carcinoma"[Title/Abstract] OR "liver cancer"[Title/Abstract] OR "HCC"[Title/Abstract] OR "liver tumor"[Title/Abstract] OR "liver neoplasms"[Title/Abstract]) AND ("Immunotherapy"[MeSH Terms] OR "Immunotherapy"[Title/Abstract] OR "antibodies monoclonal"[Title/Abstract] OR "Mab"[Title/Abstract] OR "Atezolizumab"[Title/Abstract] OR "Sintilimab"[Title/Abstract] OR "Pembrolizumab"[Title/Abstract] OR "Camrelizumab"[Title/Abstract] OR "Tremelimumab"[Title/Abstract])) NOT "Case Reports"[Publication Type]) NOT "Systematic Review"[Publication Type]) AND ((clinicaltrial[Filter]) AND (2003:2023[pdat]))	148
**2**	Web of Science	(TS=(carcinoma, hepatocellular OR Carcinomas, Hepatocellular OR Hepatocellular Carcinomas OR Liver Cell Carcinoma, Adult OR Liver Cancer, Adult OR Adult Liver Cancer OR Adult Liver Cancers OR Cancer, Adult Liver OR Cancers, Adult Liver OR Liver Cancers, Adult OR Liver Cell Carcinoma OR Carcinoma, Liver Cell OR Carcinomas, Liver Cell OR Cell Carcinoma, Liver OR Cell Carcinomas, Liver OR Liver Cell Carcinomas OR Hepatocellular Carcinoma OR Hepatoma OR Hepatomas) AND TS=(Immunotherapy OR Atezolizumab OR Sintilimab OR Pembrolizumab OR Camrelizumab OR Tremelimumab) AND TS=(randomized controlled trial OR controlled clinical trial))	324
**3**	Cochrane Library	(hepatocellular carcinoma or liver cancer or HCC or liver tumor or liver neoplasms):ti,ab,kw AND (Immunotherapy or antibodies monoclonal or Mab or Atezolizumab or Sintilimab or Pembrolizumab or Camrelizumab or Tremelimumab):ti,ab,kw (2003-2023 Filter)	1552
**4**	Embase	‘carcinoma, hepatocellular’:ab,ti OR ‘hepatocellular carcinoma’:ab,ti OR ‘liver	972

		cancer':ab,ti OR 'HCC' :ab,ti OR 'liver tumor':ab,ti OR 'liver neoplasms':ab,ti<br>'Immunotherapy':ab,ti OR 'Immunotherapy':ab,ti OR 'antibodies monoclonal':ab,ti OR 'Mab':ab,ti OR 'Atezolizumab':ab,ti OR 'Sintilimab':ab,ti OR 'Pembrolizumab':ab,ti OR 'Camrelizumab':ab,ti OR 'Tremelimumab':ab,ti (2003-2023 Filter)	

S2 Table. The commutatively comparative efficacy of different immune combination therapies.

a. Overall survival

dual immunotherapy	-	-	-
1.27 (0.67, 2.44)	immunotherapy+anti-angiogenetic agents	-	-
1 (0.55, 1.83)	0.79 (0.48, 1.28)	immunotherapy+tyrosine kinase inhibitors	-
0.78 (0.47, 1.32)	0.61 (0.42, 0.90)	0.78 (0.57, 1.06)	sorafenib

b. Progression-free survival

dual immunotherapy	-	-	-
1.49 (0.63, 3.47)	immunotherapy+anti-angiogenetic agents	-	-
1.33 (0.60, 3.02)	0.9 (0.47, 1.75)	immunotherapy+tyrosine kinase inhibitors	-
0.9 (0.45, 1.78)	0.6 (0.36, 0.99)	0.67 (0.44, 1.01)	sorafenib

c. Objective response rate

dual immunotherapy	-	-	-
1.05 (0.1, 9.91)	immunotherapy+anti-angiogenetic agents	-	-
1.58 (0.18, 12.92)	1.5 (0.27, 8.48)	immunotherapy+tyrosine kinase inhibitors	-
4.72 (0.74, 30.11)	4.49 (1.23, 17.7)	2.99 (1.04, 9.06)	sorafenib

S2 Table. The effect sizes are displayed with hazard ratio and corresponding 95% CrI. All results were presented as the ratio of the x-axis over the y-axis. (a) overall survival, (b) progression-free survival, (c) objective response rat

S3 Table. Specific treatment-related adverse events of the immune combination therapies

Specific TRAE	Immunotherapy+anti-angiogenetic agents			Immunotherapy+tyrosine kinase inhibitors			Dual immunotherapy	
	ORIENT-32 (n=380)	IMbrave-150 (n=329)	Portion （%）	LEAP-002 (n=395)	COSMIC-312 (n=429)	Portion （%）	HIMALAYA (n=393)	Portion （%）
**AST↑**	135(36)	54(16)	27	107(27)	129(30)	29	48(12)	12
**Hypertension**	121(32)	93(28)	30	238(60)	100(23)	41	23(6)	6
**Diarrhea**	55(14)	36(11)	13	187(47)	208(48)	48	103(27)	27
**Proteinuria**	160(42)	95(29)	36	141(36)	-	36	-	-
**Platelets↓**	155(41)	34(10)	27	95(24)	52(12)	18	-	-
**ALT↑**	99(26)	40(12)	20	92(23)	127(30)	27	36(9)	9
**Bilirubin↑**	112(29)	-	29	88(22)	56(13)	17	20(5)	5
**Hypothyroidism**	53(14)	33(10)	12	162(41)	89 (21)	30	47(12)	12
**Appetite↓**	43(11)	38(12)	11	127(32)	116(27)	29	66(17)	17
**Fatigue**	-	54(16)	16	116(29)	106(25)	27	66(17)	17
**PPE Syndrome**	1(＜1)	5(2)	(＜1)	143(36)	183(43)	40	3(1)	1
**Weight↓**	57(15)	-	15	88(22)	73(17)	20	-	
**Pruritus**	39(10)	47(14)	12	-	43(10)	10	89(23)	23
**Rash**	26(7)	32(10)	8	-	61(14)	14	87(22)	22

S3 Table. Data are n (%). TRAE, treatment-related adverse events; PPE syndrome, palmar-plantar erythrodysesthesia ALT, alanine aminotransferase; AST, aspartate aminotransferase; ↓ for decrease; ↑ for increase.
